# Genotype and Successive Harvests Interaction Affects Phenolic Acids and Aroma Profile of Genovese Basil for Pesto Sauce Production

**DOI:** 10.3390/foods10020278

**Published:** 2021-01-30

**Authors:** Michele Ciriello, Luigi Formisano, Christophe El-Nakhel, Marios C. Kyriacou, Georgios A. Soteriou, Fabiana Pizzolongo, Raffaele Romano, Stefania De Pascale, Youssef Rouphael

**Affiliations:** 1 Department of Agricultural Sciences, University of Naples Federico II, 80055 Portici, Italy; michele.ciriello@unina.it (M.C.); luigi.formisano3@unina.it (L.F.); christophe.elnakhel@unina.it (C.E.-N.); fabiana.pizzolongo@unina.it (F.P.); raffaele.romano@unina.it (R.R.); depascal@unina.it (S.D.P.); 2 Department of Vegetable Crops, Agricultural Research Institute, 1516 Nicosia, Cyprus; m.kyriacou@ari.gov.cy (M.C.K.); soteriou@ari.gov.cy (G.A.S.)

**Keywords:** sweet basil, Mediterranean diet, nitrate content, linalool, rosmarinic acid, chromatographic analysis, volatile compounds

## Abstract

Basil (*Ocimum basilicum* L.) is an essential ingredient of the Mediterranean cuisine due to its distinctive aroma. Genovese basil leaves are used to prepare “pesto”, a condiment that has always caught the interest of consumers and producers. Usually, basil for industrial processing is harvested more than once to extract a higher yield. However, successive cuts can affect quality traits that play a crucial role in defining the product’s final sensory profile. This research was aimed to evaluate the impact of cut on the quantitative and qualitative properties of three Genovese basil cultivars (Aroma 2, Eleonora and Italiano Classico) grown in an open field. Nitrate content, phenolic acids and aromatic profile were determined by ion chromatography (IC), high-performance liquid chromatography (HPLC), and gas chromatography coupled to a mass spectrometer (GC/MS) analysis, respectively. The second harvest increased fresh biomass and total phenolic acids content by 172% and 413%, respectively, with Italiano Classico recording the highest values. The combination of second-cut Aroma 2 yielded the lowest nitrate (473.8 mg kg^−1^ of fresh weight) and Eugenol (2.4%) levels. In the second harvest, Eleonora showed an increase in eugenol and trans-α-bergamotene of 75.3% and 48.2%, respectively; whereas, eucalyptol and β-cis-ocimene decreased by 34.4% and 51.6%, respectively. Although successive harvests may increase basil yield and quality overall, the cultivar-dependent response to successive cuts needs to be accounted for in order to accomplish standardization of industrial “pesto” sauce.

## 1. Introduction

Over the last decades, the growing interest in a healthy lifestyle has motivated consumers towards wholesome eating choices, where natural products with high nutritional value and high quality are an integral part of the daily diet [1]. There is no doubt that a balanced regime is the key to psychophysical well-being because a diet based on unhealthy nutritional models represents a high-risk factor for the onset of obesity and related diseases [2]. Moreover, recent investigations have highlighted the impact of nutrition in altering the gut microbiome, which is critical in modulating and transforming nutrient intake, with direct consequences for people’s health [3]. Since the 1950s, many studies on eating habits carried out in Mediterranean regions provided insight on the benefits of healthy and well-balanced nutrition, based on the consumption of fruit, vegetables, legumes, and cereals [4]. This led to the development of a novel nourishment model (i.e., the Mediterranean diet) declared as part of the Intangible Cultural Heritage of Humanity by UNESCO (United Nations Educational, Scientific and Cultural Organization). In the Mediterranean diet, which finds its cradle in Italy, vegetables and leafy herbs are the protagonists of every meal, providing fiber, minerals, vitamins, and antioxidants, making it popular and appreciated worldwide [4]. In traditional Mediterranean cuisine, aromatic herbs are a must-have ingredient, exalting the dishes’ organoleptic features, increasing their palatability, and providing antioxidants that assist in the reduction of chronic and cardiovascular diseases [5].

On top of that, aromatic herbs belonging to Lamiaceae are recognized as an affluent reserve of specialized metabolites that attract the interest of academic researchers [6]. Among the aromatic species, basil (*Ocimum basilicum* L.) stands out for its unique and pleasant aroma, as well as high mineral and vitamin content and low protein and lipid content [7]. Historically employed in folk medicine [8], basil is grown and marketed on a global scale [6] for its low caloric and high bioactive compounds content [9]. Its use as a fresh or dried herb made this polyhedral leafy herb ideal for seasoning soups, salads, meat, fish, and traditional Italian recipes [10]. Basil is indeed the main ingredient of the famous green sauce typical of the Riviera Ligure (Italy), known as “pesto”. Its preparation requires tender leaves of the basil cultivar Genovese D.O.P. (Protected Designation of Origin), distinguished by a high content of linalool and eugenol and the absence of estragole, which confers the unmistakable and well-appreciated flavor [11]. Basil is a functional food that draws the consumers’ and the pharmaceutical industries’ attention due to its remarkable organoleptic properties. In the last decade, the high demand for fresh basil leaves from industry and the fresh market has pushed Italian farmers to increase the cultivated area by almost 90%, with a total production of 7800 tons (ISTAT; Italian National Institute of Statistics) [12].

The richness in essential oils, which confers its distinct aroma, makes basil appreciated in gastronomy and in the pharmaceutical and cosmetic fields [13,14]. Essential oils, which are biosynthesized by specialized leaf epidermal outgrowths (i.e., glandular trichomes), belong to different classes of compounds with the most significant fraction represented by terpenes (e.g., oxygenated monoterpenes, hydrocarbon sesquiterpenes, and oxygenated sesquiterpenes) and phenylpropanoids, which have a proven antioxidant property [15]. In addition, basil is also characterized by a high phenolic content [16], providing broad-spectrum protection against chronic diseases [17]. Moreover, phenolic compounds have antifungal and antimicrobial activity [18,19] so much as to be considered multitargeting drugs [20]. High nutraceutical value is attributable to phenolics like rosmarinic, caffeic, and chicoric acids [21,22], among which the former is the most abundant in basil and has higher antioxidant activity, thus acting as a radical scavenger [23].

Considering that vegetables constitute the primary source of nitrate exposure in the human diet [24], its high content represents a critical anti-nutritional factor. Basil, like other leafy vegetables, accumulates nitrate in its tissues [25]. Although the regulation n° 1258/2011 of the European Commission has not set a threshold value for basil, its leaves can accumulate nitrate values higher than 5000 mg kg^−1^ of fresh weight [26]. Pharmacologically, nitrate has very low toxicity, but if ingested, it is reduced by saliva and digestive system into nitrite and N-nitrose compounds (e.g., nitrosamines), which oxidize hemoglobin into methemoglobin, interfering with oxygen transport and causing in children a pathology known as methemoglobinemia [27,28].

However, the high genetic variability of the genus *Ocimum* and intensive plant breeding undertaken over the years make farmers uncertain about the best suitable basil cultivar for their needs. In conventional cultivation, Genovese basil plants for “pesto” are harvested more than once during the growing season (up to three times) [10,29,30]. Taking into account that the biosynthesis of desired specialized metabolites can be stimulated by biological, physical, and chemical agents, the mechanical stress induced by successive cuts may have a considerable impact on the secondary metabolism of different genotypes [31]. Nevertheless, at present, there are no reports in the literature that unveil the cut effect on the quality traits of Genovese basil.

In this regard, our research was aimed to characterize three basil cultivars for the industrial production of “pesto Genovese” in response to two successive harvests. Production, nitrate accumulation, aromatic and phenolic profiles were evaluated. To the authors’ knowledge, this is the first research investigating these quality aspects, profiting the food industry, and paving the way for future studies.

## 2. Materials and Methods

### 2.1. Plant Material and Experimental Design

**Figure 1:**
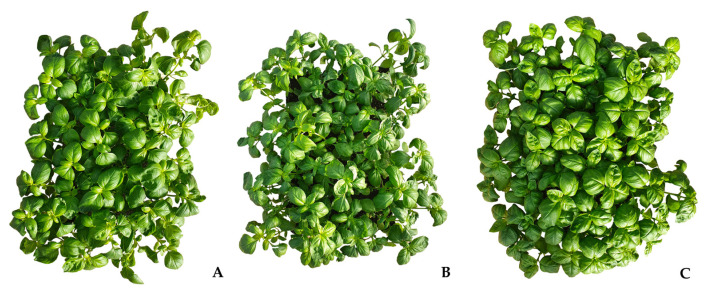
Genovese basil genotype used in the experiment. Aroma 2 (**A**), Eleonora (**B**), and Italiano Classico (**C**).

The field experiment was carried out in 2019 during the spring-summer season at the experimental site of the Department of Agriculture of the University of Naples “Federico II” in Bellizzi (Salerno, Italy). Three basil cultivars, Aroma 2 (Fenix), Eleonora (Enza Zaden), and Italiano Classico (La Semiorto) (Figure 1), were transplanted on 6 June 2019 with a density of 250 plants m^−2^. The experiment was designed as a factorial combination of three cultivars and two harvests, where the cultivars were arranged in a randomized block design, with three repetitions. Each experimental plot covered an area of 2 m^2^ and was set 0.5 m apart from the other plots. A drip irrigation system facilitated fertigation management. The experimental trial lasted 55 days, during which the two harvests (CT1 and CT2) were carried out at 34 and 55 days after transplanting (DAT), respectively. Fifty plants per plot were harvested in the pre-flowering stage, leaving two internodes to ensure an adequate vegetative regrowth. For the determination of dry weight (dw), the fresh weight of leaves and stems were recorded and then were dried to constant weight in a forced-air oven at 70 °C for about three days. Part of the fresh plant samples was immediately frozen in liquid N and then stored at −80 °C for volatiles and phenolics determination.

### 2.2. CIELAB Color Leaf Measurement

Color coordinates were recorded using a Minolta Chroma Meter CR-300 (Minolta Camera Co. Ltd., Tokyo, Japan). At each harvest date, ten measurements were taken on the upper surface of young expanded leaves of ten plants per experimental unit. As described by the International Commission of Illumination (CIE), the color was expressed with degree of lightness (L*), greenness (a*), and yellowness (b*) values, chroma, and hue angle. Chroma is the color saturation quantitative attribute representing the degree of visual difference from neutral grey of the same lightness. Higher chroma values indicate a higher color intensity perceived by humans. The hue angle describes the qualitative color attribute with respect to the red/green (+a*/−a*) and yellow/blue (+b*/−b*) axes.

### 2.3. Nitrate Content Determination

According to the method of Rouphael et al. [32], to determine the nitrate content, the dried samples were milled with a MF10.1 cutting-grinding head mill (IKA^®^, Staufen im Breisgau, Baden-Württemberg, Germany) and sieved with MF0.5 sieve (0.5 mm hole size; IKA^®^, Staufen im Breisgau, Baden-Württemberg, Germany). Fifty milliliters of purified water with Arium^®^ Advance EDI pure water system (Sartorius, Goettingen, Lower Saxony, Germany) was added to 250 mg of dry sample and then placed in a SW22 shaking water bath (Julabo, Seelbach, Baden-Württemberg, Germany) at 80 °C for 10 min and centrifuged at 6000 rpm for 10 minutes with a R-10M centrifuge (Remi Elektrotechnik Ltd., Mumbai, Maharashtra, India). The supernatant was taken, filtered using a syringe filter with a 0.45 µm pore size (Whatman International Ltd., Maidstone, Kent, UK), and analyzed by Ion Chromatography (IC). Nitrate determination was performed with an IONPAC^®^ ATC-HC 9 × 75 mm anion trap (Thermo Scientific™ Dionex™, Sunnyvale, CA, USA), an IONPAC^®^ AG11-HC 4 × 50 mm guard column (Thermo Scientific™ Dionex™, Sunnyvale, CA, USA), and an IONPAC^®^ AG11-HC 4 × 50 mm IC column (Thermo Scientific™ Dionex™, Sunnyvale, CA, USA), using a 1 mM–50 mM hydroxide gradient with a flow of 1.5 mL min^−1^. Auto Suppression Recycle Mode with a temperature of 30 °C were used. The results were obtained as g kg^−1^ dw, then based on each sample dry matter, were converted to mg kg^−1^ of fresh weight (fw).

### 2.4. Phenolic Acids: Extraction, Determination, and Quantification 

The free phenolic acids extraction and quantification (i.e., caffeic, rosmarinic, chicoric, p-Coumaric, and ferulic acids) were performed by High-Pressure Liquid Chromatography (HPLC) according to the method described by Ciriello et al. [33].

All reagents and solvents were HPLC grade (Sigma Aldrich, Milan, Italy). Two mL of 70% aqueous methanol (*v/v*) were mixed with 100 mg of freeze-dried basil leaves and then stirred for 1 minute with a Vortex Classic stirrer (Velp^®^ Scientifica, Usmate Velate, Monza Brianza, Italy), sonicated for 20 min with a Q500 ultrasonic sonicator (Qsonica, Newtown, Connecticut, USA) and shaken for 10 minutes with a SSL4 see-saw rocker (Cole-Parmer™, Vernon Hills, IL, USA). The extracts were centrifuged at 6800 rpm for 10 minutes with a R-10M centrifuge (Remi Elektrotechnik Ltd., Mumbai, Maharashtra, India) and filtered through a 0.45 μm Teflon membrane filter (Phenomenex, Torrance, CA, USA) into vials for analysis. Chromatographic separation of free phenolic compounds was performed on an Agilent Technologies Model 1100 chromatograph (Palo Alto, CA, USA) equipped with a G4225A degasser, a G13111A four-channel low-pressure gradient pump, and a G1315B diode-matrix detector, using a 20 μl sample injection volume. A reversed-phase Kinetex^®^ C18 100 Å column (5 μm particle size, 150 × 4.6 mm; Phenomenex, Torrance, CA, USA) was used. The eluents were 0.1% (*v/v*) trichloroacetic acid in water (A) and acetonitrile (B). The gradient program was 0–50% B for 50 min with a constant flow of 1 mL min^−1^. Detection of the individual free phenolic compounds was performed at 280 nm and shown in the representative chromatogram in Supplementary Figure S1.

The calibration curves were constructed using seven concentration levels (0.15, 0.5, 1, 10, 20, 50, and 100 mg L^−1^) for each standard. The concentration of each phenolic compound was reported as mean ± SE (standard error) expressed in mg kg^−1^ dw, *n* = 3.

### 2.5. Aroma Profile: Extraction and Determination

The determination of volatile compounds (VOCs) was performed by Gas Chromatography coupled to a Mass Spectrometer (GC/MS) after extraction and concentration by Solid-Phase MicroExtraction (SPME) technique [33].

For SPME, 500 mg of frozen basil leaves (−20 °C) were at, manually crushed, and placed into a 20 mL glass headspace vial with a screw cap and PTFE septum (Supelco^®^, Bellefonte, PA, USA). The vial was stirred with an ARE^®^ magnetic stirrer (Velp^®^ Scientifica, Usmate Velate, Monza Brianza, Italy) for 10 min at 30 °C to facilitate the VOCs migration into the headspace.

A 1 cm long and 50/30 μm thick divinylbenzene/carboxane/polydimethylsiloxane fiber (Supelco^®^, Bellefonte, PA, USA) was introduced into the vial to absorb VOCs and then introduced into the split-splitless injector of GC 6890N coupled to MS 5973N (Agilent, Santa Clara, CA, USA) at 230 °C for 10 min (desorption phase). The GC/MS was equipped with a 30 m long and 0.250 mm thick capillary column, coated with a 0.25 μm 5% Phenyl/95% dimethylpolysiloxane film (Supelco^®^, Bellefonte, PA, USA). Splitless injection was used for sample analysis. The oven temperature was maintained at 50 °C for 2 min and increased from 50 °C to 150 °C to 10 °C/min and from 150 °C to 280 °C to 15 °C/min. The injection source and ion source temperatures were 250 °C and 230 °C, respectively, while helium was used as a carrier gas with a flow rate of 1 mL min^−1^. The mass spectrometer was set to 70 eV.

The identification of VOCs in the headspace was performed comparing the mass spectra and retention times of the different VOCs with the Atomic Spectra Database version 1.6 (U.S. Department of Commerce, Gaithersburg, MD, USA) of the National Institute of Standards and Technology (NIST). Each treatment was analyzed in triplicate, with the corresponding results expressed as % total area normalization.

### 2.6. Statistical Analysis

A Two-way Analysis of variance (ANOVA) was implemented to assess the significance of the effects and interaction between the two main factors (Cultivar (C) and Cut (CT)). The mean effect of C and CT were compared according to One-way ANOVA and *t*-test, respectively. Statistical significance was determined at the *p* < 0.05 level using Duncan’s Multiple Range Test (DRMT) for C × CT interaction and C factor. All data are presented as mean ± standard error (SE). All statistical analyses were performed using IBM SPSS Statistics.

## 3. Results and Discussion

### 3.1. Production Response

The ample versatility of basil, used both in the culinary and cosmetic fields, has attracted the industry’s interest, which requires great amounts of fresh produce available all year round. Inevitably, this pattern has driven growers to select cultivars with higher productivity and to adopt agricultural practices to maximize crop yield.

**Figure 2:**
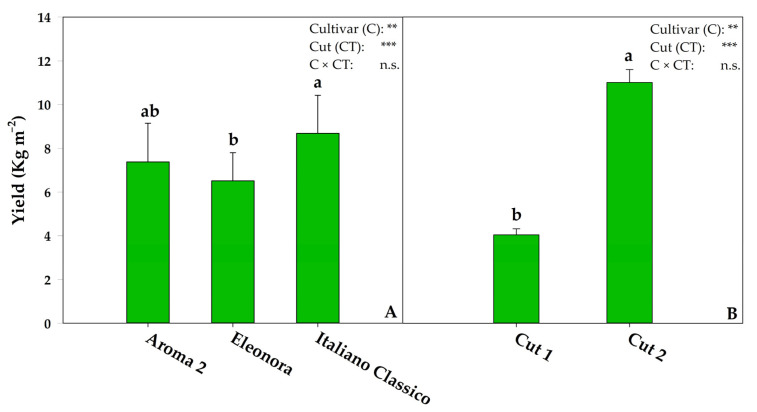
Effect of cultivar (**A**) and cut (**B**) on the yield of green Genovese basil. n.s., **, *** non-significant or significant at *p* ≤ 0.01 and 0.001, respectively. Cultivar means were compared by ANOVA; according to Duncan’s multiple range test (*p* = 0.05), different letters indicate significant differences. Cut means were compared using a *t*-test. All data are expressed as mean ± standard error (SE) (*n* = 3).

In the current experiment, the interaction of both factors, cultivar and cut (C × CT), did not result in significant variations in fresh biomass production, in contrast to a significant effect of cultivar and cut factors (Figure 2). Concerning the cultivar, the average total fresh biomass was 7.53 kg m^−2^; this result, in accordance with the typical values of intensive cultivation of basil for industrial processing, which is probably due to the high plant density [34]. However, among the tested cultivars, Italiano Classico had the highest unit production (8.69 kg m^−2^), higher by 18% and 33% than Aroma 2 and Eleonora, respectively, although it was only statistically significant in respect to the latter (Figure 2). The results confirmed the significant genotypic impact on total production, as demonstrated by other recent research on basil [35].

Similar to other leafy vegetables (e.g., rocket, spinach, coriander), basil is harvested more than once in a crop cycle to ensure the highest yield and to amortize the costs of production [10,34]. In our experiment, the two successive cuts resulted in notable differences in the total production of fresh biomass (Figure 2). Specifically, the yield of the second cut was 172% higher than the first, in agreement with previous findings [30,36]. As stated by Zheljazkov et al. [36], the higher production achieved in the second cut could be due to a well-rooted hypogeal system, which limits the competition for nutrients and water uptake, thus helping to reconstitute the epigeal system efficiently. A further study suggested that, in response to the cut, the interruption of apical dominance reduced the auxinic flow, which would promote the lateral shoot emission as occurred in our experiment (data not shown), leading to an increase in production [30]. However, a recent investigation on basil grown in a soilless system does not corroborate the findings presented in the current experiment, as it demonstrated a reduction in fresh biomass at the second cut [10].

### 3.2. Leaf Colorimetry

Visual quality is undoubtedly of crucial relevance, which can influence the industry and consumer choice [37]. However, the color of plants varies according to genetic and pre-/post-harvest factors, such as agronomic practices, maturation, and storage methods [38]. The characteristic green color of basil leaves is an important industrial requirement for the preparation of “pesto Genovese”. A more intense color attracts the consumer’s interest and reduces the use of artificial colorants.

The color of the leaves was affected by genotype and cut (Table 1); however, the interaction of these factors was not significant for color coordinates L*, a*, and b* and for chroma and hue angle. The b* values demonstrated a cultivar-dependent variation, with Italiano Classico and Eleonora recording the highest (21.62) and lowest (18.64) ones, respectively. These results are consistent with the chroma values that indicate a lower color intensity perceived in Eleonora, although statistically not different from Aroma 2. On the other hand, the degree of lightness (L*) and greenness (a*) did not vary, which means that the leaves maintained the same shade of green in each cultivar. These findings are in accordance with those reported in a comparable study on green basil [39], which showed that, under the same growing conditions, plants tended to change mainly the b* value, without affecting the a* value, confirming that this effect depends only on the genotype. Notwithstanding the change in color from green to yellow is usually attributed to tissue senescence [40], in this experiment basil plants were harvested in the pre-flowering stage, without senescence symptoms, as evidenced by the high yield achieved, highlighting the influence of the genotype once again.

The cut mean effect shows that the second harvest positively impacted the colorimetric parameters (Table 1). L* and a* values decreased by 3.5% and 11.4%, respectively. These results are supported by the hue angle, which decreased by 2.1%, showing the change in leaf coloring towards green. The freshly harvested vegetables usually have a glossy and bright surface (higher L* value), which generally varies with post-harvest processing [38]. However, in our experiment, the lower gloss and the greater shade of green could be due to a higher accumulation of antioxidants in response to mechanical stress induced by the cut for protecting the photosynthetic machinery and preserving it from oxidation. In fact, an intense green color indicates a more significant accumulation of compounds with antioxidant function [41], confirmed by the increased build-up of phenolic acids achieved at the second cut, in accordance with our results.

### 3.3. Nitrate Accumulation

**Figure 3:**
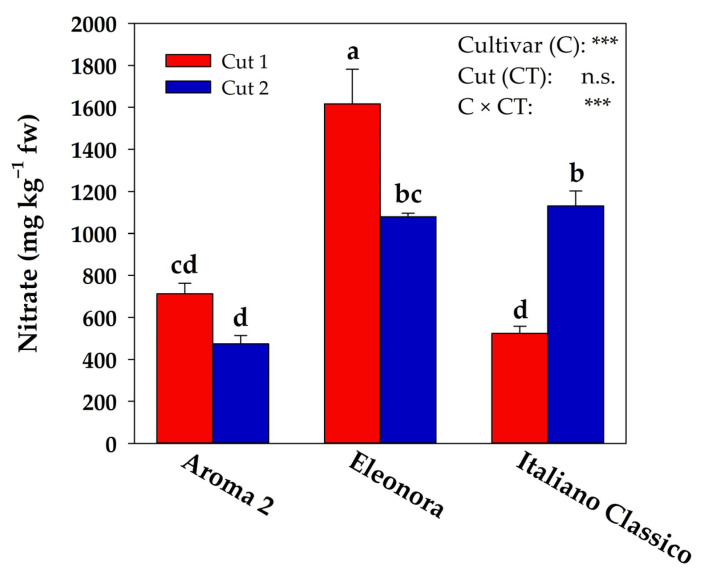
Nitrate content in different cultivars and cuts. n.s. and *** non-significant or significant at *p* ≤ 0.001, respectively. According to Duncan’s multiple range test (*p* = 0.05), different letters indicate significant differences.

The nitrate content was affected by the cultivar and cut interaction (Figure 3). Aroma 2 and Eleonora recorded, on average, a decreased nitrate content of ~33% in the second cut; in contrast, Italiano Classico showed an opposite trend with a higher nitrate concentration of 1130.97 mg kg^−1^ fw (+116%) in the second harvest.

Nitrate is the primary source of nitrogen for plants that are accumulated in considerable amounts in their tissues. However, scientific evidence regarding the correlation of nitrate consumption with chronic disease onset relegates it among the most widely recognized anti-nutritional compounds [25]. Usually, nitrate is safe due to its rapid excretion in the urine [42], but under specific conditions, it can be reduced to nitrite [43], which is acknowledged to play a crucial role in carcinogenesis and the incidence of blood diseases such as methemoglobinemia [27]. Given its well-documented hazardousness, nitrate is a potentially harmful anion that can affect horticultural produce quality since its raw consumption is the principal source of human dietary intake [26].

However, in our experiment, Eleonora recorded, on average, 95% more nitrate than Aroma 2 and Italiano Classico (Figure 3), under the same growing conditions (spring-summer and high light intensity), thus evidencing a clear genotypic impact, in accordance with former studies [26,42]. To corroborate our results, it is worth pointing out that only Aroma 2 and Eleonora showed a lower nitrate content at the second harvest (Figure 3). This finding was also achieved by Nicoletto et al. [30], underlining further that the effect of cut-induced mechanical stress could be genotype-dependent. The investigation carried out by Corrado et al. [10] gave an additional explanation for the increase in nitrate in reply to the cut, tracing it back to passive plasticity that can determine plant luxuriation. However, contrary to our experiment, the fact that only one cultivar of basil had been used cannot exclude a priori the genotype effect.

Nevertheless, in the present experiment, the tested Genovese basil cultivars accumulated low nitrate values (473.78–1616.41 mg kg^−1^ fw) compared to similar results achieved in a comparable study performed in soilless systems under a protected environment [42]. Apart from the genetic aspect, according to Santamaria [44], the environment plays a vital task in nitrate build-up. Especially, the nitrate reductase enzyme in highlight conditions has higher activity, leading to a lower nitrate accumulation in plant tissues. Despite its potentially high nitrate content, basil is not yet regulated by the European Commission regulation n 1258/2011. However, even the highest value presently achieved by Eleonora at the first cut (Figure 3) is below the maximum threshold set for spring-summer leafy vegetables.

### 3.4. Impact of Cultivar and Cut on Phenolic Acids Accumulation

Due to their sessile nature, plants rely on effective defensive systems to protect themselves against potential environmental threats. Among passive protection mechanisms, specialized metabolites play a relevant role in plant survival and colonization of our planet [45]. Most of the technological and nutritional attributes of medicinal herbs such as basil are indeed associated with their high levels of these metabolites, of which phenolic acids are the most representative [8].

The HPLC assay of the phenolic profile in the experimented basil plants revealed a predominance of rosmarinic, chicoric, and caffeic acids, in accordance with the results achieved by Prinsi et al. [46]. Apart from p-Coumaric acid, the phenolic profile was affected by the interaction of the examined factors (Table 2). In the second harvest, there was a considerable rise in phenolic acids concentration in all the assessed cultivars. Notably, Italiano Classico recorded the highest increase of chicoric acid (517%) and rosmarinic acid (1128%), whereas caffeic and ferulic acids increase was higher in Aroma 2, marking 237% and 162%, respectively. Eleonora recorded the highest value of p-Coumaric acid, rising by 160% in the second cut (Table 2). Compared with other cultivars, Italiano Classico showed the highest total phenolic acids content (1080.79 mg kg^−1^ dw), mainly due to the high rosmarinic acid (Table 2). This finding demonstrates a strong genotypic impact of basil on the biosynthesis of the above-mentioned specialized metabolites [45,47]. It also points out the tendency of Genovese cultivars to metabolize preferably rosmarinic acid [30]. According to Nicoletto et al. [30], rosmarinic acid increased after the cut, independently from the cultivar. This result confirms that the stress induced by the cut can be a valuable tool to increase the antioxidant activity of basil. Chemically, rosmarinic acid is the ester of caffeic acid, belonging to the chemical group of phenylpropanoids, whose biosynthesis occurs via the amino acids L-tyrosine and L-phenylalanine pathway [48,49]. Its molecular structure, characterized by the presence of hydroxyl groups, confers relevant antioxidant activity and a regulating function on tyrosinase enzyme activity and melanin production [50,51], which provide benefits in the prevention of disease, including diabetes and skin melanoma [23]. It could also be a valuable adjuvant in the development of new antibiotic drugs with its strong and recognized antibiotic activity [52]. The amount of rosmarinic acid obtained in this study did not reflect the data in the literature, which could be due to the different growing conditions as well as the analytical method used to assess them, as suggested by Maggini et al. [53]. In our samples, rosmarinic acid ranged from 87.98 to 1185.29 mg kg^−1^ dw, in contrast to the results recorded in previous studies [47,54]. The results achieved by Javanmardi et al. [54] showed rosmarinic acid values up to 100 times higher than those obtained in our investigation. However, it is worth noting that non-domesticated basil plants used by Javanmardi et al. [54] were obtained from unselected seeds from local markets or supplied by farmers. As argued by Vallarino et al. [55], domestication would determine a reduction in secondary metabolic activity in favor of the primary one, supporting the results obtained in our work.

The phenolic compounds in aromatic herbs have a high antioxidant activity that imparts health benefits, reinforces the immune system, and improves life expectancy [5]. These bioactive compounds’ attributes are a valuable resource for the food industry to replace the widespread synthetic antioxidants (e.g., BHA, butylated hydroxyanisole; BHT, Butylated hydroxytoluene) [8], thus making the food system safer and more sustainable. For example, Genovese basil with high phenolic acid content could be an excellent tool to improve “pesto” quality, extending its shelf-life and reducing oxidation during storage [56]. As the build-up of polyphenols is an adaptive plant response to adverse environmental conditions [57], resulting in higher oxygen reactive species (ROS) evolution [58,59]. The increase in polyphenols in all basil cultivars in response to subsequent cuts, reveals that this agricultural practice is a helpful tool to grow basil with better biochemical features.

Despite the application of eustress that enhances the concentrations of desirable phytochemicals [45] but often results in a slowdown in growth rates [60], our findings showed a surprising increase in unit yield caused by the cut. As suggested by Crozier et al. [61] and Shaw et al. [62], the improvement in production performance and the consequent increase in photosynthetic products may have enhanced the phenolic concentrations because of the allocation of excessively fixed carbon to the shikimate pathway

### 3.5. Impact of Cultivar and Cut on Aroma Profile

In Genovese basil for “pesto” production, aroma is undoubtedly the critical quality attribute for taste determination [63]. The unmistakable intense aroma of Genovese basil, without mint flavor, is characterized by wide variability in the composition of essential oils, of which monoterpenes and phenylpropanoids are the major constituents [34].

SPME-GC/MS analysis of the volatiles of all tested basil cultivars identified 40 molecules, among which six were above 2% (Table 3). The most abundant compound was lalool, followed by eucalyptol, trans-α-bergamotene, eugenol, 1-octen-3-ol, and β-cis-ocimene. The interaction between the factors under investigation showed significant changes in the aroma profile, except for linalool. The eucalyptol content recorded a substantial decrease of 34.4% in Eleonora in response to the cut. Similarly, β-cis-ocimene showed the same trend in Italiano Classico (−24.1%) and Eleonora (−51.6%), while in Aroma 2 it was unchanged. On the contrary, in Eleonora, eugenol, and trans-α-bergamotene increased in the second harvest by 75% and 48.2%, respectively, whereas the highest value of 1-octen-3-ol was achieved in the first harvest. However, in comparison with Aroma 2 and Italiano Classico, the aroma profile of Eleonora was characterized by a reduced linalool content. 

**Figure 4:**
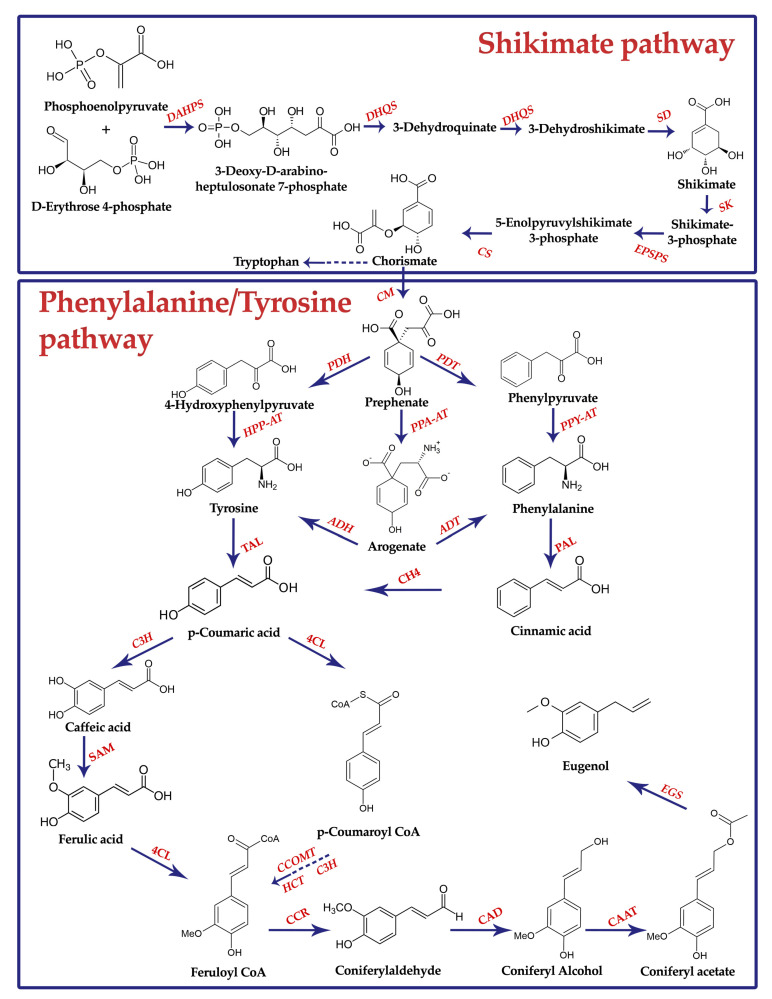
Aromatic amino acid biosynthesis in plants: schematic diagram of the shikimate and phenylalanine/tyrosine pathways. Dashed arrows indicate different enzymatic steps. Abbreviations: DAHPS, 3-Deoxy-d-arabino-2-heptulosonate 7-phosphate synthase; DHQS, 3-Dehydroquinate synthase; DHQD, 3-Dehydroquinate dehydratase; SD, Shikimate dehydrogenase; SK, Shikimate kinase; EPSPS, 5-Enolpyruvylshikimate 3-phosphate synthase; CS, Chorismate synthase; CM, Chorismate mutase; PDH, Prephenate dehydrogenase; PDT, Prephenate dehydratase; HPP-AT, 4-Hydroxyphenylpyruvate aminotransferase; PPY-AT, Phenylpyruvate aminotransferase; PPA-AT, Prephenate aminotransferase; ADH, Arogenate dehydrogenase; ADT, Arogenate dehydratase; TAL, Tyrosine ammonia-lyase; C4H, Cinnamate 4-hydroxylase; PAL, Phenylalanine ammonia-lyase; C3H, p-Coumarate 3-hydroxylase; 4CL, 4-Hydroxycinnamoyl CoA ligase; SAM, S-Adenosylmethionine synthetase; CCOMT, Caffeoyl-CoA O-methyltransferase; HCT, Hydroxycinnamoyl-CoA shikimate/quinate hydroxycinnamoyl transferase; CCR, Cinnamoyl reductase; CAD, Cinnamyl alcohol dehydrogenase; CAAT, Coniferyl alcohol acetyltransferase; EGS, Eugenol synthase.

Regarding the average cut impact, linalool increased in the second harvest by 25.8%. The aroma profile results are in accordance with similar research findings, where linalool predominated while estragole was absent; the latter compound is neither appreciated by consumers nor by the food industry [11,34]. It is worth noting that linalool, other than its role in enhancing oil quality, also has a well-documented anxiolytic and anti-depressive properties, which can be exploited as a supplementary therapeutic to alleviate the symptoms of these diseases that afflict a large part of the population [64]. However, each volatile compound’s biosynthesis showed significant variation among the sampled cultivars, highlighting how genetic variables impact the aroma profile [65]. Interestingly, eugenol content showed a robust cultivar-dependent response, with the maximum value recorded in Italiano Classico (6.68%), which was also characterized by the highest total phenolic acids’ build-up. Indeed, eugenol is a phenylpropanoid that, like other phenolic acids, shares the same metabolic pathway [66] (Figure 4). Moreover, several studies pointed to eugenol as the key volatile compound with antioxidant activity in basil [67,68].

Our findings revealed a high impact of the cut on the expression of the oxygenated monoterpenes (Table 3), thus supporting that the biosynthesis of these specialized metabolites depends on genetic and stress-related factors [69]. In our study, linalool increased in response to the cut, in agreement with several authors [29,36] compared with the other oxygenated monoterpenes, which showed an opposite trend. Although linalool, eucalyptol, and β-cis-ocimene share the same precursor (GPP, geranyl pyrophosphate), it is still unclear how both environmental and cut factors may affect gene expression of linalool synthase (LIS) enzyme activity, 1,8-cineole synthase, and β-cis-ocimene synthase that respectively catalyze the conversion of GPP to linalool, eucalyptol, and β-cis-ocimene [70].

In aromatic herbs such as basil, the genotype × environment interaction mostly affects the biosynthesis of specialized metabolites and volatile oils concentration [71], in part confirming our results. Specifically, we observed a more robust cultivar-dependent response in Eleonora, which after harvesting, a −34.4%, −51.6%, and +75.3% variation in eucalyptol, β-cis-ocimene, and eugenol content, respectively, was evidenced. The simultaneous decrease of the monoterpenes (eucalyptol and β-cis-ocimene) and the increase of the phenylpropanoid eugenol, which are synthesized via two distinct metabolic pathways (i.e., mevalonate and shikimate pathways) [72], confirm that terpene synthase is negatively correlated with phenylalanine ammonia-lyase (PAL) activity [73].

## 4. Conclusions

For the food industry, the final product’s standardization is the pivotal goal for its commercial success, increasingly focused on quality requirements. Aroma and color constitute outstanding and influential basil quality indicators to consumers. As stated in this study, the cut affected all these aspects, posing a challenge to the processing industry, which must guarantee uniformity in production. Therefore, it must be considered in order to identify the best Genovese basil cultivar for this purpose. The findings achieved for the aromatic profile showed a cultivar-dependent response to the cuts. Specifically, Aroma 2 and Italiano Classico underwent a lower variation in volatiles than Eleonora, in conformity to the agro-industry demands. All cultivars reacted positively to the cuts, resulting in better productive performance (+172%) as well as in the bioaccumulation of specialized metabolites (+413%) to which are attributed beneficial health properties that draw the appreciation of the food, cosmetic and pharmaceutical industries. Finally, for “pesto” basil cultivation, Aroma 2 showed the best performance in response to cuts by achieving high yield, standard sensory profile, and the lowest nitrate content.

## Figures and Tables

**Table 1:** Effect of cultivar and cut on CIELAB colorimetric coordinates, chroma, and hue angle of basil leaves.

Source of Variance	L *			a *			b *				Chroma				Hue Angle		
Cultivar (C)																	
Aroma 2	45.80	±	0.91	−7.31	±	0.29	20.06	±	0.66	ab	21.36	±	0.71	ab	110.10	±	0.37
Eleonora	45.59	±	0.73	−6.85	±	0.35	18.64	±	0.57	b	19.89	±	0.60	b	110.32	±	0.87
Italiano Classico	45.40	±	0.64	−7.59	±	0.22	21.62	±	0.42	a	22.93	±	0.45	a	109.49	±	0.46
	n.s.			n.s.			*				*				n.s.		
Cut (CT)																	
CT 1	46.41	±	0.66	−6.81	±	0.23	20.00	±	0.62		21.14	±	0.66		108.84	±	0.25
CT 2	44.79	±	0.38	−7.69	±	0.17	20.20	±	0.61		21.64	±	0.62		111.09	±	0.33
*t*-test	*			**			n.s.				n.s.				***		
C × CT																	
Aroma 2 × CT1	46.74	±	1.62	−6.97	±	0.35	19.96	±	1.08		21.15	±	1.13		109.34	±	0.22
Aroma 2 × CT2	44.86	±	0.78	−7.65	±	0.43	20.15	±	1.00		21.56	±	1.08		110.86	±	0.22
Eleonora × CT1	47.02	±	0.73	−6.21	±	0.45	18.48	±	0.95		19.52	±	1.04		108.65	±	0.60
Eleonora × CT2	44.16	±	0.26	−7.49	±	0.09	18.79	±	0.82		20.25	±	0.77		111.98	±	0.81
Italiano Classico × CT1	45.46	±	1.16	−7.24	±	0.09	21.57	±	0.45		22.77	±	0.43		108.54	±	0.39
Italiano Classico × CT2	45.34	±	0.84	−7.94	±	0.34	21.67	±	0.83		23.10	±	0.89		110.43	±	0.04
	n.s.			n.s.			n.s.				n.s.				n.s.		

n.s., *, **, and *** non-significant or significant at *p* ≤ 0.05, 0.01, and 0.001, respectively. According to Duncan’s multiple range test (*p* = 0.05), different letters indicate significant differences. Cut means were compared by a *t*-test. All data are expressed as mean ± SE (n = 3). L *: lightness; a *: greenness; b *: yellowness.

**Table 2:** Effect of cultivar and cut on free phenolic acids (mg kg^−1^ dw) profile of basil.

Source of Variance	Caffeic Acid				Chicoric Acid				Rosmarinic Acid				*p*-Coumaric Acid				Ferulic Acid				Total Phenolic Acids			
Cultivar (C)																								
Aroma 2	95.86	±	23.35	b	226.56	±	75.18	b	420.48	±	149.27	b	6.63	±	1.32	ab	17.24	±	3.48		766.77	±	252.23	b
Eleonora	111.07	±	6.73	a	304.53	±	72.07	a	366.19	±	99.41	b	7.04	±	1.41	a	16.83	±	1.67		805.67	±	179.73	b
Italiano Classico	90.62	±	20.40	b	326.47	±	105.50	a	640.91	±	244.14	a	6.24	±	1.25	b	16.54	±	1.44		1080.79	±	371.94	a
	***				***				***				*				n.s.				***			
Cut (CT)																								
1	62.13	±	8.86		98.81	±	12.49		111.64	±	10.03		3.69	±	0.11		12.3	±	0.77		288.55	±	31.33	
2	136.24	±	4.07		472.90	±	26.72		840.09	±	92.19		9.59	±	0.21		21.44	±	1.13		1480.27	±	112.92	
*t*-test	***				***				***				***				***				***			
C × CT																								
Aroma 2 × CT1	43.88	±	1.81	d	59.96	±	3.02	d	87.98	±	3.88	d	3.69	±	0.30		9.51	±	0.24	c	205.03	±	4.05	d
Aroma 2 × CT2	147.83	±	4.71	a	393.17	±	22.21	b	752.98	±	28.89	b	9.56	±	0.30		24.96	±	0.92	a	1328.50	±	50.46	b
Eleonora × CT1	97.45	±	0.38	c	145.22	±	1.12	c	150.39	±	6.34	d	3.90	±	0.11		13.88	±	0.62	c	410.83	±	5.95	c
Eleonora × CT2	124.70	±	6.37	b	463.85	±	24.31	b	582.00	±	52.93	c	10.18	±	0.10		19.79	±	2.18	ab	1200.51	±	74.70	b
Italiano Classico × CT1	45.05	±	1.51	d	91.24	±	0.99	cd	96.54	±	2.54	d	3.47	±	0.12		13.5	±	0.90	c	249.79	±	2.90	cd
Italiano Classico × CT2	136.20	±	1.37	ab	561.70	±	17.92	a	1185.29	±	40.91	a	9.02	±	0.30		19.58	±	0.56	b	1911.79	±	33.48	a
	***				***				***				n.s.				**				***			

n.s., *, **, and *** non-significant or significant at *p* ≤ 0.05, 0.01, and 0.001, respectively. According to Duncan’s multiple range test (*p* = 0.05) different letters indicate significant differences. Cut means were compared by a *t*-test. All data are expressed as mean ± SE (*n* = 3).

**Table 3:** Effect of cultivar and cut on aroma profile (%) of basil.

Source of Variance	1-octen-3-ol				Eucalyptol				β-cis-ocimene				Linalool				Eugenol				Trans-α- Bergamotene			
Cultivar (C)																								
Aroma 2	3.64	±	0.19	b	27.11	±	1.64		2.90	±	0.13	b	39.62	±	1.65	a	2.89	±	0.42	c	3.55	±	0.54	b
Eleonora	4.48	±	0.29	a	27.47	±	2.86		3.73	±	0.59	a	35.47	±	2.65	b	4.63	±	0.59	b	6.95	±	0.70	a
Italiano Classico	3.13	±	0.16	b	24.17	±	0.96		3.21	±	0.22	b	39.81	±	1.94	a	6.68	±	0.59	a	6.00	±	0.46	a
	***				n.s.				**				**				***				***			
Cut (CT)																								
1	3.85	±	0.35		28.16	±	1.61		3.84	±	0.33		33.92	±	1.27		4.78	±	0.79		5.55	±	0.38	
2	3.65	±	0.11		24.34	±	1.40		2.72	±	0.13		42.68	±	0.46		4.68	±	0.59		5.45	±	0.88	
*t*-test	n.s.				*				***				***				n.s.				n.s.			
C × CT																								
Aroma 2 × CT1	3.67	±	0.39	b	25.42	±	2.09	ab	2.83	±	0.09	bc	36.29	±	1.49		3.32	±	0.73	bc	4.51	±	0.47	bc
Aroma 2 × CT2	3.61	±	0.19	b	28.80	±	2.49	ab	2.96	±	0.27	bc	42.95	±	0.50		2.45	±	0.38	c	2.59	±	0.55	c
Eleonora × CT1	5.02	±	0.34	a	33.18	±	2.62	a	5.02	±	0.16	a	29.66	±	1.04		3.36	±	0.27	bc	5.60	±	0.28	b
Eleonora × CT2	3.95	±	0.11	ab	21.75	±	1.12	b	2.43	±	0.15	c	41.28	±	0.52		5.89	±	0.24	a	8.30	±	0.76	a
Italiano Classico × CT1	2.86	±	0.22	b	25.86	±	0.83	ab	3.65	±	0.15	b	35.80	±	1.56		7.67	±	0.76	a	6.55	±	0.61	ab
Italiano Classico × CT2	3.40	±	0.10	b	22.48	±	1.02	b	2.77	±	0.16	c	43.82	±	0.59		5.69	±	0.47	ab	5.46	±	0.61	b
	*				**				***				n.s.				**				**			

n.s., *, **, and *** non-significant or significant at *p* ≤ 0.05, 0.01, and 0.001, respectively. According to Duncan’s multiple range test (*p* = 0.05) different letters indicate significant differences. Cut means were compared by a *t*-test. All data are expressed as mean ± SE (*n* = 3).

## Data Availability

The datasets generated for this study are available on request to the corresponding author.

## Supplementary Materials

The following are available online at https://www.mdpi.com/2304-8158/10/2/278/s1. Figure S1. Separation of caffeic acid (1), p-Coumaric acid (2), ferulic acid (3), chicoric acid (4), and rosmarinic acid (5) in Genovese basil extract by HPLC.
