# Supplementary material for: Genotype and Successive Harvests Interaction Affects Phenolic Acids and Aroma Profile of Genovese Basil for Pesto Sauce Production
Source: Foods. 2021 Jan 30;10(2):278. doi: 10.3390/foods10020278 (PMC7911349; doi:10.3390/foods10020278)
Supplement: Supplementary file 1 [file foods-10-00278-s001.pdf]

foods
MDPI

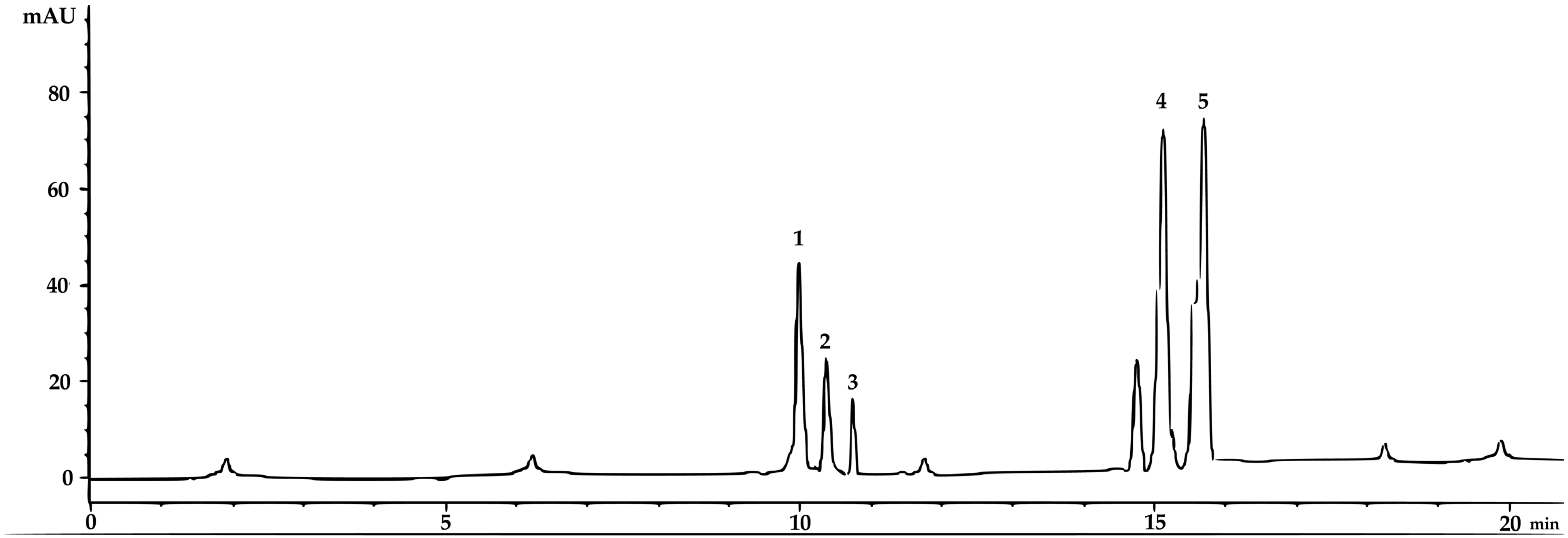

Figure S1. Separation of caffeic acid (1), p-Coumaric acid (2), ferulic acid (3), chicoric acid (4), and rosmarinic acid (5) in Genovese basil extract by HPLC.
